# Cyclophilin A/CD147 Interaction: A Promising Target for Anticancer Therapy

**DOI:** 10.3390/ijms23169341

**Published:** 2022-08-19

**Authors:** Jang Mi Han, Hye Jin Jung

**Affiliations:** 1 Department of Life Science and Biochemical Engineering, Graduate School, Sun Moon University, Asan 31460, Korea; 2 Department of Pharmaceutical Engineering and Biotechnology, Sun Moon University, Asan 31460, Korea; 3 Genome-Based BioIT Convergence Institute, Sun Moon University, Asan 31460, Korea

**Keywords:** anticancer therapy, cyclophilin A, CD147, *PPIA*, *BSG*

## Abstract

Cyclophilin A (CypA), which has peptidyl–prolyl *cis-trans* isomerase (PPIase) activity, regulates multiple functions of cells by binding to its extracellular receptor CD147. The CypA/CD147 interaction plays a crucial role in the progression of several diseases, including inflammatory diseases, coronavirus infection, and cancer, by activating CD147-mediated intracellular downstream signaling pathways. Many studies have identified CypA and CD147 as potential therapeutic targets for cancer. Their overexpression promotes growth, metastasis, therapeutic resistance, and the stem-like properties of cancer cells and is related to the poor prognosis of patients with cancer. This review aims to understand the biology and interaction of CypA and CD147 and to review the roles of the CypA/CD147 interaction in cancer pathology and the therapeutic potential of targeting the CypA/CD147 axis. To validate the clinical significance of the CypA/CD147 interaction, we analyzed the expression levels of *PPIA* and *BSG* genes encoding CypA and CD147, respectively, in a wide range of tumor types using The Cancer Genome Atlas (TCGA) database. We observed a significant association between *PPIA/BSG* overexpression and poor prognosis, such as a low survival rate and high cancer stage, in several tumor types. Furthermore, the expression of *PPIA* and *BSG* was positively correlated in many cancers. Therefore, this review supports the hypothesis that targeting the CypA/CD147 interaction may improve treatment outcomes for patients with cancer.

## 1. Introduction

Cancer threatens human life, and diverse cancer therapies, including surgery, radiation therapy, chemotherapy, immunotherapy, and targeted therapy, have been developed [1]. However, existing cancer therapies are only effective for some malignant tumors and frequently result in treatment failure in patients with advanced cancer [2]. The failure of cancer treatment is mainly attributed to cancer metastasis, recurrence, heterogeneity, resistance to chemotherapy and radiation therapy, and immune evasion [3]. Recently, cancer stem cells (CSCs), a subgroup of cancer cells with stem-like characteristics, such as self-renewal ability and multi-lineage differentiation, have been recognized as critical contributors to treatment failure [4,5,6]. Thus, the eradication of CSCs may improve clinical outcomes in patients with cancer [7]. Owing to the efforts to develop effective cancer treatments, many studies have discovered several biomarkers and signaling pathways that play vital roles in upregulating the malignant features of cancer cells, including proliferation, anti-apoptosis, invasion, angiogenesis, therapeutic resistance, and stemness.

Cyclophilin A (CypA) is a cytoplasmic protein belonging to the peptidyl–prolyl isomerase (PPIase) family, which regulates various biological functions by binding to its receptor, CD147 [8]. Accumulating evidence has revealed that the CypA/CD147 interaction is implicated in several diseases, including inflammatory diseases, coronavirus infection, and cancer [8,9]. CypA and CD147 are potential therapeutic targets for cancer because they are central drivers of tumor progression and poor prognosis [10,11,12]. They are overexpressed in many cancer tissues, and cellular responses stimulated by their interactions are closely associated with cancer malignancy [8,13]. Activation of the CypA/CD147 axis induces the proliferation, metastasis, and drug resistance of cancer cells as well as increases the survival of CSCs [10,11,12,13,14,15,16].

In this review, we focus on the role of CypA/CD147 interactions in cancer development and the therapeutic potential of targeting them. In addition, based on a pan-cancer analysis of The Cancer Genome Atlas (TCGA) data, we explore the clinical significance of CypA/CD147 interactions in various tumor types.

## 2. Biological Functions of CypA and CD147

### 2.1. Biology of CypA

**Figure 1:**
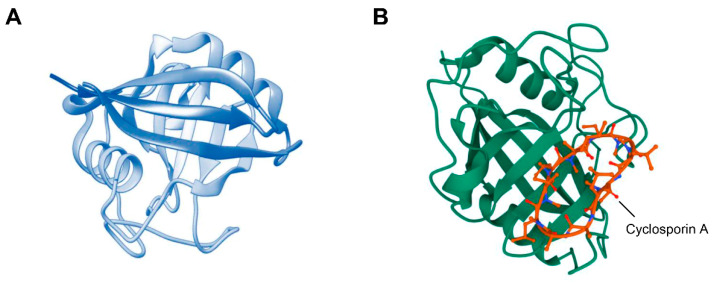
Structure of CypA. (**A**) CypA has an 8-stranded antiparallel β-barrel structure with two α helices surrounding the barrel from either side. (**B**) Structure of CypA-CsA complex. CsA binds to the PPIase active site of CypA.

Cyclophilins are an evolutionarily conserved and ubiquitous family of proteins in all prokaryotes and eukaryotes [17]. They are peptidyl–prolyl isomerases (PPIases) that catalyze the isomerization of the peptide bond from the *trans*-form to the *cis*-form at the proline residue, promoting protein folding as a molecular chaperone [18,19]. Cyclophilin A (CypA) is the most abundant member of the cyclophilin family and has an eight-stranded antiparallel β-barrel structure with two α-helices surrounding the barrel from either side [17] (Figure 1A). CypA was first identified as a target protein for the immunosuppressant cyclosporin A (CsA), which binds to the active site of CypA, interfering with its PPIase activity [20] (Figure 1B). CypA exists in the cytoplasm but can be secreted from cells by proinflammatory stimuli, such as hypoxia and oxidative stress, in an autocrine or paracrine manner [21,22,23]. Secreted CypA mediates various intercellular communication and intracellular responses [21]. CypA acts as a proinflammatory cytokine in endothelial cells and stimulates the growth of muscle cells [24,25]. In addition, CypA promotes the formation and infectivity of HIV-1 virions, positive regulation of the Th1 profile for T-cell activation, and suppression of Th2 differentiation [26,27,28]. CypA is involved in oxidative stress-mediated neurodegenerative diseases, rheumatoid arthritis, sepsis, and aging [29,30,31,32]. CypA alters the tumor microenvironment by promoting the development of Th1 immune responses through modulation of matrix metalloproteinase (MMP) and induction of proinflammatory cytokines such as tumor necrosis factor alpha (TNFα) and interferon gamma (IFNγ) and thus may control the early stages of tumor and metastasis formation [33]. CypA is overexpressed in many cancers and promotes cancer cell proliferation, anti-apoptosis, and metastasis/infiltration [34,35,36]. Therefore, CypA has multifunctional physiological properties within cells and plays a pathological role in the development of inflammatory diseases and cancer.

### 2.2. Biology of CD147

**Figure 2:**
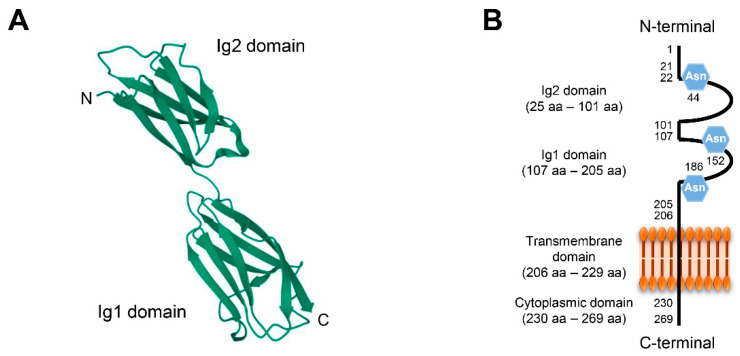
Structure of CD147. (**A**) Structure of extracellular Ig domains of CD147. (**B**) One monomer of CD147 is 269 amino acids (aa) in length and consists of two extracellular Ig domains, Ig1 and Ig2, a single transmembrane domain, and a short cytoplasmic domain. The extracellular region of CD147 contains three asparagine (Asn) glycosylation sites.

CD147, also called basigin or EMMPRIN, is a glycosylated transmembrane protein that belongs to the immunoglobulin (Ig) superfamily [37]. It is abundant on the surface of various types of tumors and stromal cells and was initially discovered as a tumor-cell-derived collagenase-stimulating factor [38]. Recent studies have revealed its role as a MMP inducer and tumor promotor [39,40]. Structurally, one monomer of CD147 consists of two extracellular Ig domains, Ig1 and Ig2; a single transmembrane domain; and a short cytoplasmic domain (Figure 2A,B). CD147 monomers can homodimerize in a *cis*-dependent manner at the plasma membrane, interact with different cells in a *trans*-dependent manner, and induce intracellular signaling [41,42]. Soluble forms of CD147 can be internalized, leading to cell proliferation, metastasis, and invasion [43,44]. Accumulating evidence has demonstrated that CD147 is a potential target for preventing and treating several diseases [15,44,45,46]. The inhibition of CD147 expression alleviated atherosclerosis and exhibited therapeutic effects in collagen-induced arthritis [45,46]. CD147 reportedly serves as a receptor for severe acute respiratory syndrome coronavirus 2 (SARS-CoV-2) [47]. Furthermore, CD147 is overexpressed in various cancer cells and promotes the proliferation, metastasis, angiogenesis, and stemness of cancer cells through the activation of major oncogenic signaling pathways [15,44,48,49]. These findings reveal the potential of CD147 as a biomarker or therapeutic target for various human diseases, including cancer.

### 2.3. Interaction between CypA and CD147

**Figure 3:**
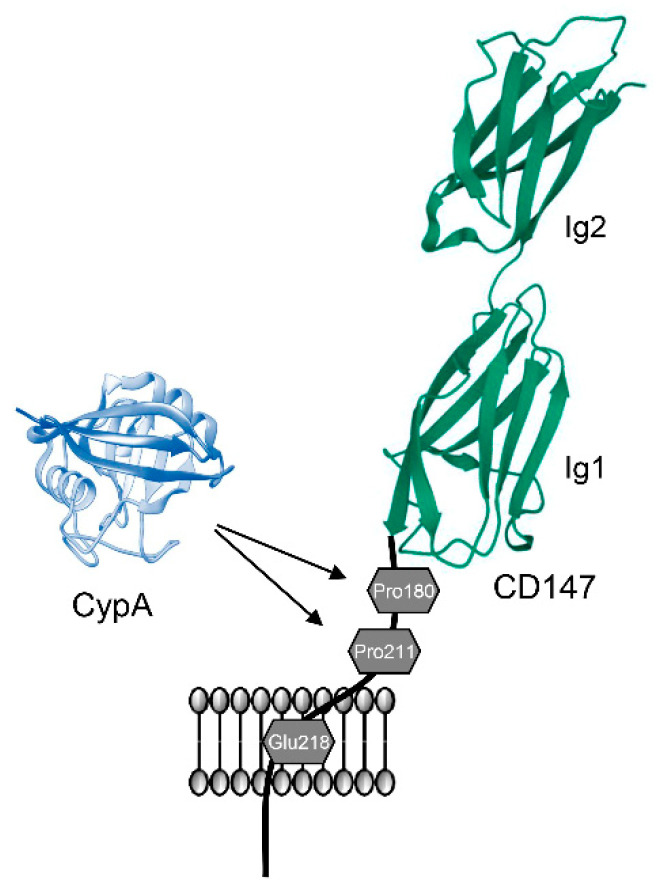
Interaction between CypA and CD147. CypA binds to amino acid Pro^180^ of CD147 and induces signal transduction through subsequent interaction with Pro^211^. The amino acid Glu^218^ of CD147 is also important for the signaling response.

CypA interacts with the cellular receptor CD147 to exert multiple functions through cellular signaling cascades [50]. Genetic analysis demonstrated that CypA binds to the amino acid Pro^180^ of CD147 and induces signal transduction through subsequent interaction with Pro^211^ and that the amino acid Glu^218^ is vital for the signaling responses [51] (Figure 3). CypA-mediated isomerization of CD147 stabilized the *cis*-conformer of the Trp^210^-Pro^211^ peptidyl-prolyl bond, resulting in a “proline switch” [52]. More recently, it has been suggested that the CypA/CD147 binding process may be regulated by Pro^180^-Gly^181^ rather than Pro^211^ and that the amino acid Arg^201^ is important for the binding [53]. Thus, the interaction between CypA and CD147 may represent a ligand-receptor interaction, in which proline isomerization outside the cell results in intracellular signaling [52]. Recent studies have demonstrated the critical role of CypA/CD147 interactions in tumor development [8,13]. Inhibition of CypA suppressed proliferation and increased apoptosis of gastric cancer cells by downregulating the expression of CD147 and CD147-mediated downstream signaling pathways, indicating that the CypA/CD147 interaction plays a vital role in cancer cell proliferation [13]. Moreover, CD147 expression was consistently upregulated in tumors with increased CypA expression, suggesting that the interaction between CypA and CD147 may activate cancer pathogenesis [8].

## 3. Roles of CypA/CD147 Interaction in Cancer

### 3.1. Proliferation

**Figure 4:**
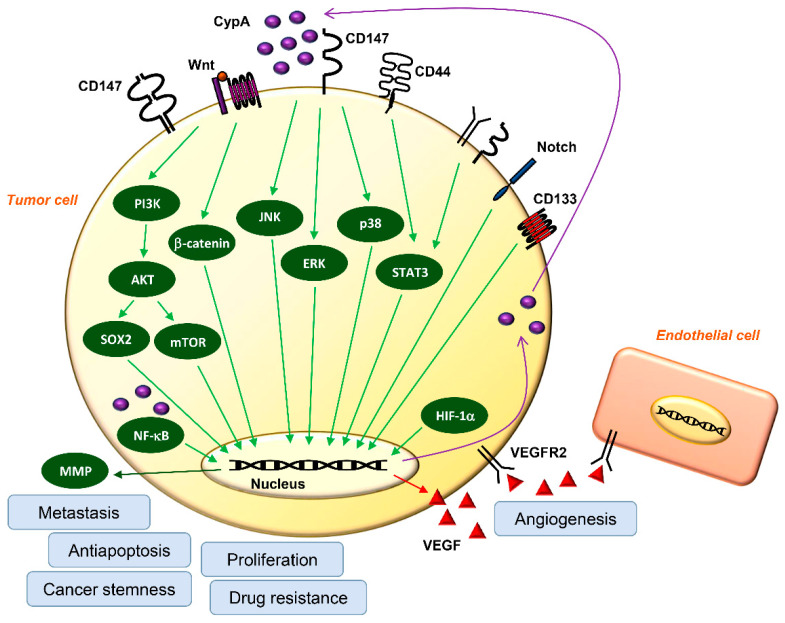
Major oncogenic signaling pathways regulated by CypA/CD147 axis. CypA expression can be regulated by HIF-1α and interacts with its receptor CD147 through autocrine/paracrine extracellular secretion. The CypA/CD147 interaction can activate directly or indirectly multiple oncogenic signaling pathways, including PI3K/AKT, Wnt/β-catenin, MAPKs, STAT3, Notch, and NF-κB, thereby promoting proliferation, antiapoptosis, metastasis, angiogenesis, drug resistance, and stemness of cancer cells.

The roles of CypA and CD147 in cancer development were studied (Figure 4 and Table 1). They are upregulated in various types of cancers and have been identified as crucial mediators of tumorigenesis and cancer cell proliferation [33,54,55,56,57,58]. Hypoxia-inducible factor (HIF)-1α, activated by hypoxia, can promote the expression and autocrine secretion of CypA, leading to cancer growth [36,56]. Overexpression of CypA in small-cell lung cancer increases cell growth by activating extracellular signal-regulated protein kinase 1/2 (ERK1/2) signaling, whereas CypA silencing inhibits growth [34,54]. In 68% of liver fluke-associated cholangiocarcinoma (CCA), overexpression of CypA enhanced proliferation, but its inhibition significantly suppressed the proliferation of CCA cell lines [57]. Notably, neither CypA silencing nor overexpression affected the proliferation of non-tumor human cholangiocytes [57]. Moreover, CypA and CD147 were overexpressed in mycosis fungoides/Sézary syndrome (MF/SS) tumor cells, and inhibition of CypA and CD147 suppressed the proliferation of cutaneous T-cell lymphoma cell lines both in vitro and in vivo by downregulating the phosphorylation of ERK1/2 and protein kinase B (AKT) [59]. CypA and CD147 were expressed at relatively higher levels in pancreatic cancer cell lines than in normal human pancreatic ductal epithelial cells [58]. Consistently, their expression levels were higher in human pancreatic adenocarcinoma tissues than in normal pancreatic tissues [58]. The addition of exogenous CypA stimulates the proliferation of pancreatic cancer cells by activating the ERK1/2 and p38 mitogen-activated protein kinase (MAPK) signaling pathways and by increasing the secretion of interleukin (IL)-5 and IL-17. In contrast, the effect is blocked through CD147 inhibition [58]. CypA also increased the stability of nuclear factor kappa B (NF-κB) p65, promoting its nuclear translocation and accumulation [34]. Consequently, the activated transcription factor NF-κB induces IL-8-mediated cancer cell proliferation [60,61]. Moreover, CypA promotes cell proliferation by inducing cell cycle transition from G1 to S phase in hepatocellular carcinoma (HCC) [62]. The expression of CypA is significantly higher in stages III and IV HCC than in stages I and II at the tumor, node, and metastasis stage, suggesting that CypA overexpression causes higher tumor malignancy [62]. Recently, inhibition of CypA was shown to significantly suppress the proliferation of gastric cancer cells by downregulating the CD147-mediated phosphatidylinositol-3-kinase (PI3K)/AKT/mammalian target of rapamycin (mTOR), c-Jun N-terminal kinase (JNK), and ERK1/2 pathways [13,54,60,61]. Notably, FK506-binding protein (FKBP), a PPIase family member like CypA, has a negative correlation with the survival of patients with lung cancer and promotes cancer growth through PPIase activity [63]. In addition, overexpression of FKBP correlates with poor prognosis in patients with glioma, whereas FKBP knockdown inhibits glioma growth in vivo [64]. Furthermore, the isomerase activity of CypA is essential for tumorigenesis and the proliferation of estrogen receptor (ER)+ and ER− breast cancers through Janus kinase 2 (JAK2)/signal transducers and activators of transcription 5 (STAT5) activation, implying that PPIase activity is essential for cancer cell proliferation [65]. In another study, CypA/CD147 interactions stimulated the expression of cyclin D1 and survivin through phosphorylation of STAT3. They co-localized with the cancer stem cell marker CD44, promoting tumorigenesis and the growth of pancreatic cancer [66]. Moreover, CD147 overexpression in bladder cancer increases the expression of cell proliferation antigen Ki-67 and promotes cell proliferation. However, patients with CD147 overexpression have a poor prognosis [67]. These findings suggest that CypA/CD147 interactions may act as a major intracellular signaling mediator for cancer cell proliferation.

### 3.2. Metastasis

CypA and CD147 are involved in cancer metastasis and invasion [68,69,70,71,72,73]. CypA promotes proliferation and metastasis of non-small-cell lung cancer through the activation of p38 MAPK [68]. The CypA-overexpressing group had a relatively higher rate of lung tumor metastasis than the CypA-downregulated group in the mouse model [68]. In addition, CypA and MMP expression was positively correlated in patients with esophageal squamous cell carcinoma [69]. CD147 also induces the expression of MMP-9, and its blockade inhibits the invasion and metastasis of malignant melanoma [70,71]. Furthermore, CD147 is closely related to CD44, which enhances the metastatic ability and chemoresistance of prostate cancer [72]. Local recurrence and distant metastasis frequently occur in patients with gastric cancer with high CypA expression [73]. Inhibition of CypA/CD147 interactions effectively suppressed the metastasis and invasion of gastric cancer cells by reducing MMP-2 and MMP-9 expression [13]. Moreover, CD147 can induce angiogenesis, which plays an important role in the invasion and metastasis of malignant tumors by increasing vascular endothelial growth factor (VEGF) production, leading to a poor prognosis [74]. Therefore, the upregulation of CypA and CD147 is associated with a short survival rate in patients with metastatic cancer, and CypA/CD147 interactions may play a vital role in cancer metastasis.

### 3.3. Antiapoptosis

Activation of the CypA/CD147 interaction inhibits the apoptosis of cancer cells [13,54,75,76,77,78,79,80,81]. The antiapoptotic effects are associated with the upregulation of the PI3K/AKT/mTOR signaling pathway, modulation of the Bcl-2 family, and inhibition of caspase cascades [13,54,78,79]. Overexpression of CypA prevents hypoxia- and cisplatin-induced apoptosis in several cancer cell lines, including prostate cancer cells [56]. CypA is significantly upregulated after radiation therapy in lung adenocarcinoma cells, and CypA inhibition greatly increases radiosensitivity, reinforcing cell apoptosis [80]. CD147 is highly expressed in human epidermal growth factor receptor 2 (HER2)-positive breast cancer tissues, and its suppression enhances the anticancer efficacy of trastuzumab by increasing apoptosis in breast cancer cells [81]. Inhibition of CypA by CsA treatment also induces cell death in breast cancer cells [82]. In addition, downregulation of the CypA/CD147 axis abates cancer aggression by inducing apoptosis in glioma and gastric cancer [83,84]. These findings suggest that CypA/CD147 interaction may be a key target for regulating cancer cell apoptosis.

### 3.4. Resistance to Chemotherapy and Radiation Therapy

Interaction between CypA and CD147 can result in resistance to chemotherapy and radiation therapy [56,85,86,87]. CypA overexpression reduces cisplatin-induced apoptosis, whereas CypA silencing suppresses cancer cell viability, demonstrating that CypA upregulation can induce drug resistance [56]. In addition, CypA overexpression upregulates drug resistance-associated genes, such as IL-6, multidrug resistance-associated protein 2 (MRP2), microsomal glutathione transferase 1 (MGST1), and glutathione S-transferase zeta 1 (GSTZ1), by increasing the expression of ATP-binding cassette (ABC) transporters [85]. CypA also mediates the chemoresistance of colorectal cancer through redox modifications [86]. CD147 induces resistance to radiation therapy in hepatocellular carcinoma by interacting with integrin β1 [87]. Furthermore, CD147 stimulates the production of hyaluronic acid (HA), which promotes tumor chemotolerance by interacting with CD44 and HA receptors in prostate cancer [72]. Notably, FKBP9, which has PPIase activity similar to that of CypA, confers glioblastoma resistance to apoptosis caused by ER stress inducers [64]. These results suggest that the upregulation of the CypA/CD147 axis contributes to chemoresistance and radioresistance, leading to poor outcomes in patients with cancer.

### 3.5. Cancer Stem Cells (CSCs)

CypA and CD147 play a crucial role in the initiation, growth, and survival of CSCs [15,85,86,87]. They survive and maintain CSCs by activating the phosphatase and tensin homolog (PTEN)/PI3K/AKT signaling pathway, which is important for maintaining the CD44+/CD133+ cancer stem cell phenotype [88]. In addition, CypA/CD147 activation induces CSC features in breast cancer cells through STAT3 signaling [15]. CypA also promotes self-renewal, proliferation, and radiotherapy resistance in glioma stem cells by modulating Wnt/β-catenin signaling [14]. Notably, the gene encoding CypA was the most stably expressed essential gene in the CSC phenotype [89]. Furthermore, peptidyl–prolyl isomerase increases sphere formation, self-renewal, and metastasis of CSCs by activating Notch signaling [90]. CD147 promotes the release of small extracellular vesicles during the differentiation of colon CSCs, which can induce invasion [49]. In addition, CD147+ breast cancer cells have characteristics similar to those of breast CSCs, including self-renewal capacity, differentiation, and in vivo tumorigenic potential [91]. Solute carrier family 34 member 2 (SLC34A2)/PI3K/AKT/SRY-box transcription factor 2 (SOX2) signaling is essential for maintaining CD147+ breast CSCs [91]. Therefore, the CypA/CD147 axis may be a potential target for CSC eradication.

**Table 1:** The roles of CypA/CD147 in cancer.

The Key Processes of Cancer Progression	Protein	The Roles of CypA and CD147 in Cancer	References
Proliferation	CypA	Overexpression and autocrine secretion by activation of HIF-1α	[36,56]
		IL-8 mediated proliferation by stabilization and nuclear accumulation of NF-κB p65	[34,61]
		Promotion of the cell cycle transition from G1 to S phase	[62]
		Causes of higher tumor malignancy	[62]
		Activation of JAK2/STAT5 signaling pathway	[65]
	CD147	Promotion of tumorigenesis with CD44	[66]
		Induction of Ki-67 expression	[67]
	CypA and CD147	Activation of JNK/ERK1/2/p38 MAPK signaling pathways	[34,54,58,59,60]
		Overexpression in human tumor tissues than in normal tissues	[58]
		Induction of the secretion of IL-5 and IL-17	[58]
		Activation of PI3K/AKT/mTOR signaling pathway	[13,54,59,60]
		Poor prognosis	[63,64,67]
		Stimulation of cyclin D1/survivin by activation of STAT3	[66]
Metastasis	CypA	Induction of migration through p38 MAPK activation	[68]
		Positive correlation with MMP	[69]
		Local recurrence and distant metastasis	[73]
	CD147	Promotion of metastasis with CD44	[72]
		Induction of angiogenesis by increasing VEGF production	[74]
	CypA and CD147	Promotion of the invasion and migration by induction of MMP-2/MMP-9 expression	[13,69,70,71]
Antiapoptosis	CypA	Inhibition of hypoxia- and cisplatin-induced apoptosis	[56]
		Upregulation after radiation therapy	[80]
	CD147	Promotion of anticancer efficacy by trastuzumab	[81]
	CypA and CD147	Activation of PI3K/AKT/mTOR signaling pathway	[13,78,79]
		Modulation of Bcl-2 family	[13,78,79]
		Inhibition of caspase cascades	[13,78,79]
		Contribution to cancer aggressiveness	[83,84]
Resistance to chemotherapy and radiation therapy	CypA	Inhibition of cisplatin-induced apoptosis and causes of drug resistance	[56]
		Upregulation of IL6, MRP2, MGST1, and GSTZ1 by increasing the expression of ABC transporter	[85]
		Causes of chemoresistance through redox modification	[86]
		Causes of resistance to ER stress inducer-caused apoptosis	[64]
	CD147	Causes of radiation resistance by interacting with integrin β1	[87]
		Promotion of tumor chemotolerance through interactions with CD44 and HA receptor	[70]
Cancer stem cells	CypA	Promotion of self-renewal, proliferation, and radiotherapy resistance through Wnt/β-catenin signaling	[14]
		The most stably expressed essential gene in CSC	[89]
		Induction of sphere formation, self-renewal, and metastasis through Notch signaling	[90]
	CD147	Release of small extracellular vesicles for invasion	[49]
		Induction of self-renewal capacity, differentiation, and in vivo tumorigenic potential	[91]
		Activation of SLC34A2/PI3K/AKT/SOX2 signaling	[91]
	CypA and CD147	Promotion of CD44+/CD133+ CSCs through the activation of PTEN/PI3K/AKT	[88]
		Induction of CSC features through STAT3 signaling	[15]

## 4. Therapeutic Potential of Targeting CypA/CD147 in Cancer

Because of the considerable evidence that the interaction between CypA and CD147 plays a central role in cancer pathogenesis, they are attractive targets for the development of cancer treatment. Agents that potentially interfere with CypA/CD147 interaction can be classified as (1) drugs targeting either CypA or CD147 protein or (2) their gene-silencing drugs (Table 2).

Cyclosporin A (CsA) is the best-studied CypA inhibitor in the literature and has a wide range of biological activities, including immunosuppressive, anti-inflammatory, antifungal, and antitumor effects [20]. CsA binds to both extracellular and intracellular CypA and inhibits PPIase activity. CsA also inhibits CypA/CD147-mediated downstream signaling and cellular functions by interrupting CypA binding to CD147 [51]. CsA suppresses cancer cell growth by inducing apoptosis via a caspase-dependent pathway in several types of cancers, including breast cancer and lung adenocarcinoma [77,82]. Additionally, sanglifehrin A (SfA), a novel immunosuppressive natural product, binds to CypA with approximately 60 times higher affinity than CsA and inhibits cell proliferation by blocking the G1/S transition [92,93,94]. However, CypA inhibitors that possess immunosuppressive activity result in chronic nephrotoxicity; therefore, drugs that selectively inhibit CypA without inducing side effects have been developed [95]. The CypA inhibitor Debio-025 (Alisporivir) is a non-immunosuppressive analog of CsA and exhibits potent antitumor and antimetastatic activity by inhibiting the Crk signaling pathway in breast cancer [96]. Moreover, Debio-025 enhances tumor immunogenicity and thus improves the tumor response to anti-PD-1 therapy [96]. Other non-immunosuppressive CypA inhibitors, NIM811 and SCY-635, inhibit hepatocarcinogenesis by disrupting CypA-NS5A interaction in hepatitis C virus-induced hepatocellular carcinoma [97]. Recently, a novel CypA inhibitor, NV651, was demonstrated to be more potent than CsA and SfA in reducing the PPIase activity of cyclophilins with no immunosuppressive effect [98]. NV651 significantly inhibits cancer cell proliferation and tumor growth in hepatocellular carcinoma in vivo [98]. In addition, 23-demethyl 8,13-deoxynargenicin (compound 9) is a promising anticancer agent that targets the CypA/CD147 interaction [13]. Compound 9 is a new analog of nargenicin A1, an antibacterial macrolide with effective activity against various Gram-positive bacteria [83]. It possesses potential antitumor and antiangiogenic activity, unlike nargenicin A1 [83,99]. Proteomics analysis and further functional studies demonstrated that compound 9 binds to CypA and downregulates the CD147-mediated MAPK signaling pathway, including JNK and ERK1/2, by inhibiting CypA and CD147 expression in gastric cancer cells [13]. As a result, compound 9 suppressed the proliferation, migration, invasion, and angiogenesis of gastric cancer cells [13]. Melittin, a polypeptide containing 26 amino acid residues, also inhibits the invasion of breast cancer cells by downregulating CD147 and MMP-9 by inhibiting CypA expression [100].

The small molecule AC-73 is the first specific inhibitor of CD147 that disrupts CD147 dimerization [101]. AC-73 inhibited hepatocellular carcinoma metastasis by reducing MMP-2 production by blocking the CD147-stimulated MAPK/STAT3 signaling pathway [101]. In addition, CD147 inhibition by AC-73 resulted in a potent growth inhibitory effect in leukemia cells by deactivating the ERK/STAT3 pathway and activating autophagy, as well as increasing the chemosensitivity of leukemia cells to the conventional antileukemia drugs arabinosylcytosine and arsenic trioxide [102]. In addition to the small-molecule compound targeting CD147, the anti-CD147 drug metuximab (Licartin) prevents tumor recurrence after orthotopic liver transplantation or percutaneous radiofrequency ablation in patients with advanced hepatocellular carcinoma [103,104]. Furthermore, metuximab sensitized pancreatic cancer cells to chemoradiotherapy by reducing the CSC subpopulation by blocking CD44/STAT3 signaling [48].

In contrast, therapeutics that can induce the gene silencing of CypA or CD147 have been applied for cancer treatment. RNA interference (RNAi)-mediated gene silencing of CypA decreases proliferation and increases radiosensitivity of lung adenocarcinoma cells [54,80]. CypA knockdown inhibited glioblastoma growth by blocking NF-κB signaling [61]. In addition, inhibition of CD147 by RNAi suppresses the proliferation and invasion of colorectal CSCs and enhances chemosensitivity through the inhibition of stemness markers [105]. Moreover, silencing of the CD147 gene promoted the anticancer activity of trastuzumab by activating caspase-3/9 and deactivating MAPK and AKT signaling in HER2-positive breast cancer cells [81]. CD147 silencing also induces apoptosis through the inhibition of the X-linked inhibitor of apoptosis (XIAP) in multidrug-resistant cancer cells [76]. Several studies have revealed that miRNAs play an important role in tumorigenesis by regulating mRNA expression. miR-890, which negatively regulates the expression of CD147 mRNA, induces apoptosis and inhibits the invasion of triple-negative breast cancer cells by activating caspase-3 and decreasing MMP-9 levels [75]. Therefore, drugs that target CypA/CD147 interaction can ameliorate the growth, metastasis, and chemo/radioresistance of cancer.

**Table 2:** Therapeutic potential of targeting CypA/CD147 in cancer.

Target Protein	Inhibitors	Mechanism	Therapeutic Potential	Cancer	References
CypA	Cyclosporin A	PPIase activity	Interference of CypA and CD147 binding, Induction of apoptosis	Breast cancer, Lung adenocarcinoma	[20,51,77,82]
	Sanglifehrin A	PPIase activity	Binding to CypA with about 60-fold higher affinity than CsA	T cells, B cells, Glioblastoma multiforme	[92,93,94]
	Debio-025 (Alisporivir)	Crk signaling	Nonimmunosuppressive analogue of CsA, Potent antitumor and antimetastatic activity, Enhancing of tumor immunogenicity and anti-PD-1 therapy	Breast cancer, Hepatitis C-hepatocellular carcinoma	[96]
	SCY-635, NIM811	NS5A	Nonimmunosuppressive activity, Inhibition of hepatocarcinogenesis	Hepatitis C-hepatocellular carcinoma	[97]
	NV651	PPIase activity	Nonimmunosuppressive activity, More potent PPIase activity than CsA and SfA, Inhibition of cell proliferation and tumor growth in vivo	Hepatocellular carcinoma	[98]
	23-demethyl 8,13-deoxynargenicin (compound 9)	MAPK signaling	Inhibition of proliferation, migration, invasion, and angiogenesis	Gastric cancer	[13,83,99]
	Melittin	MMP-9	Inhibition of Metastasis	Breast cancer	[100]
	RNA interference	PPIase activity	Inhibition of tumor growth, Enhancing of radiosensitivity	Lung adenocarcinoma	[54,80]
		NF-κB signaling	Inhibition of glioblastoma growth	Glioblastoma	[61]
CD147	AC-73	CD147 dimerization, MAPK/STAT3 signaling, MMP-2	Inhibition of metastasis and growth, Activation of autophagy, Increase of chemotherapy sensitivity	Hepatocellular carcinoma, Acute myeloid leukemia	[101,102]
	Metuximab (Licartin)	CD44/STAT3 signaling	Prevention of tumor recurrence, Increase of sensitivity to chemoradiation therapy	Hepatocellular carcinoma, Pancreatic cancer	[48,103,104]
	RNA interference	Stemness markers	Suppression of the proliferation and invasion of CSCs, Promotion of chemosensitivity	Colorectal adenocarcinoma	[105]
		Caspase-3/9, MAPK pathway, AKT pathway	Promotion of anticancer activity of trastuzumab	HER2-positive breast cancer	[81]
		XIAP	Induction of apoptosis	Oral squamous carcinoma, Multidrug-resistant cancer	[76]
		Caspase-3, MMP-9	Induction of apoptosis, Inhibition of invasion	Triple-negative breast cancer	[75]

## 5. Expression and Clinical Significance of CypA and CD147 in Cancer

CypA and CD147 are encoded by the *PPIA* and *BSG* genes, respectively [106]. The genes are amplified in various tumor types, including gastric, liver, lung, pancreatic, breast, colon, and skin cancers, and their overexpression is associated with poor prognosis in patients with cancer [10,13,15,34,48,62,69,86]. Analysis of TCGA and Gene Expression Omnibus (GEO) datasets revealed that the increased expression of CypA is correlated with reduced overall survival in patients with colon, liver, or breast cancer [86,96,98]. In addition, meta-analysis of the correlation between CD147 expression and tumor prognosis revealed that elevated CD147 expression is closely related to poor survival in patients with cancer [44,107]. Therefore, CypA and CD147 are potential prognostic biomarkers and promising therapeutic targets against cancer.

**Figure 5:**
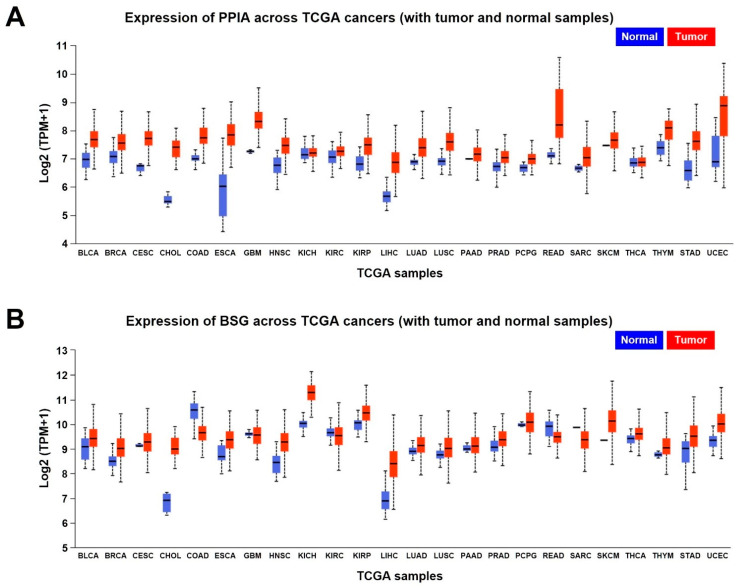
mRNA expression levels of *PPIA* and *BSG* in tumor tissues compared with normal tissues. (**A**) Expression levels of *PPIA*. (**B**) Expression levels of *BSG*. The data were obtained through UALCAN analysis using the TCGA database. BLCA, bladder carcinoma; BRCA, breast invasive carcinoma; CESC, cervical squamous cell carcinoma and endocervical adenocarcinoma; CHOL, cholangiocarcinoma; COAD, colon adenocarcinoma; ESCA, esophageal carcinoma; GBM, glioblastoma; HNSC, head and neck squamous cell carcinoma; KICH, kidney chromophobe; KIRC, kidney renal clear-cell carcinoma; KIRP, kidney renal papillary cell carcinoma; LIHC, liver hepatocellular carcinoma; LUAD, lung adenocarcinoma; LUSC, lung squamous cell carcinoma; PAAD, pancreatic adenocarcinoma; PRAD, prostate adenocarcinoma; PCPG, pheochromocytoma and paraganglioma; READ, rectal adenocarcinoma; SARC, sarcoma; SKCM, skin cutaneous melanoma; THCA, thyroid carcinoma; THYM, thymoma; STAD, stomach adenocarcinoma; UCEC, uterine corpus endometrial carcinoma.

To further validate the expression and clinical significance of the CypA/CD147 axis in a wide range of cancers, we performed gene expression and survival analyses of *PPIA* and *BSG* in 24 different tumor types. Analysis of TCGA gene expression data using UALCAN revealed that the expression levels of *PPIA* were upregulated in all 24 tumor tissues compared with those in normal tissues (Figure 5A, Table 3). Among the 24 tumor types, *PPIA* expression is markedly higher in cholangiocarcinoma, esophageal carcinoma, and uterine corpus endometrial carcinoma than in normal tissues. The expression levels of *BSG* were also upregulated in most tumor tissues compared with normal tissues but downregulated in colon adenocarcinoma, glioblastoma, kidney renal clear-cell carcinoma, rectal adenocarcinoma, and sarcoma (Figure 5B, Table 4).

**Figure 6:**
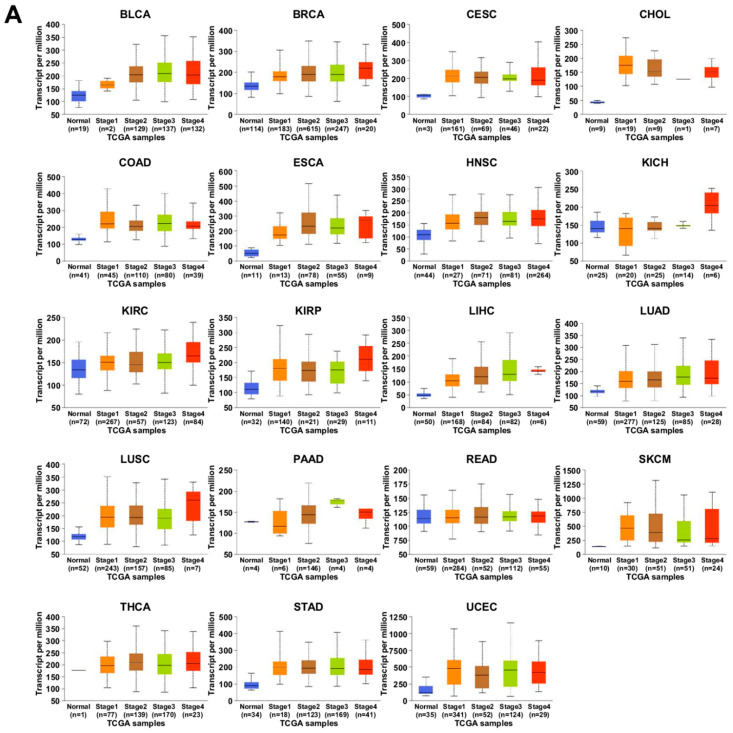

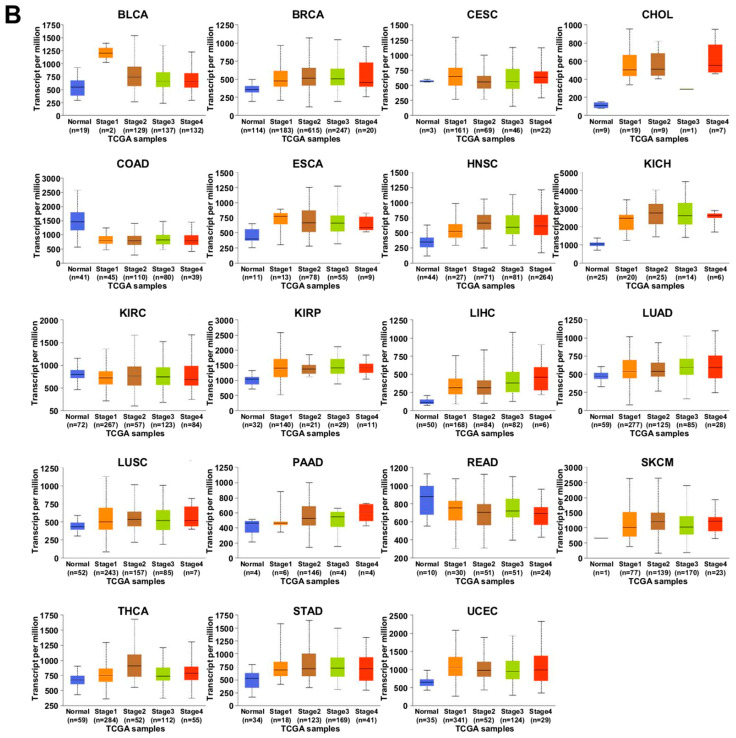
Transcription levels of *PPIA* and *BSG* in individual cancer stages (normal, stage 1, stage 2, stage 3, stage 4). (**A**) Expression levels of *PPIA*. (**B**) Expression levels of *BSG*. The data were obtained through UALCAN analysis using the TCGA database. The full name of each carcinoma is described in the legend in Figure 5.

Subsequently, we analyzed the relationship between the cancer stage and the expression levels of *PPIA* and *BSG* using UALCAN. Unfortunately, the expression levels in glioblastoma, prostate adenocarcinoma, pheochromocytoma, paraganglioma, sarcoma, and thymoma were unavailable. Most cancers have four stages: stage (1) cancer is small and has not spread anywhere else, stage (2) cancer has grown but has not spread, stage (3) cancer is larger and may have spread to the surrounding tissues and/or the lymph nodes, and stage (4) cancer has spread from where it started to at least one other body organ and is also known as “secondary” or “metastatic” cancer [108]. The expression levels of *PPIA* increased with increasing cancer stage in bladder carcinoma, breast invasive carcinoma, esophageal carcinoma, kidney chromophobe, kidney renal clear-cell carcinoma, kidney renal papillary cell carcinoma, liver hepatocellular carcinoma, lung adenocarcinoma, and lung squamous cell carcinoma (Figure 6A). In contrast, *BSG* expression levels were higher at cancer stages 3 and 4 than at cancer stages 1 and 2 in liver hepatocellular carcinoma, lung adenocarcinoma, and pancreatic adenocarcinoma (Figure 6B). Notably, the expression levels of both *PPIA* and *BSG* were increased at advanced cancer stages in liver hepatocellular carcinoma and lung adenocarcinoma, indicating that the overexpression of CypA/CD147 may be associated with the poor prognosis of patients with liver or lung cancer.

Interestingly, lung squamous cell carcinoma showed a different expression pattern from lung adenocarcinoma. In lung adenocarcinoma, the expression levels of *PPIA* and *BSG* gradually increased until cancer stage 3, but there was no further increase in cancer stage 4. In contrast, in lung squamous cell carcinoma, the expression of *PPIA* further increased at cancer stage 4, whereas the expression of *BSG* was similar at all cancer stages. These results suggest that the upregulation of *PPIA* expression, but not *BSG*, may be associated with the promotion of cancer metastasis in lung squamous cell carcinoma. However, lung adenocarcinoma showed a similar expression pattern of between *PPIA* and *BSG* in all cancer stages, indicating that targeting CypA/CD147 interactions may be more effective for the treatment of lung adenocarcinoma than lung squamous cell carcinoma.

**Figure 7:**
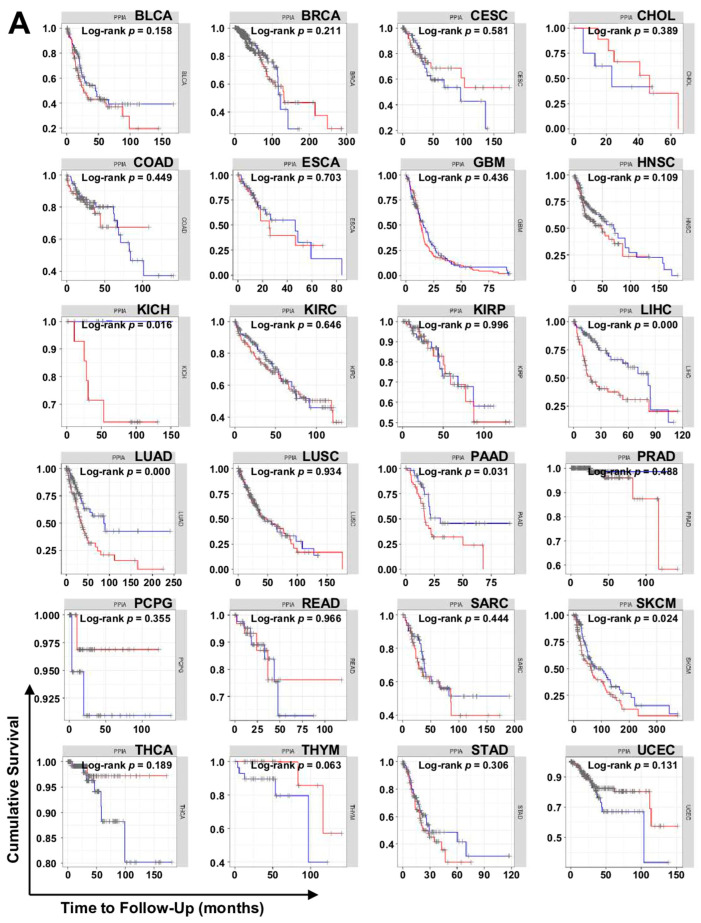

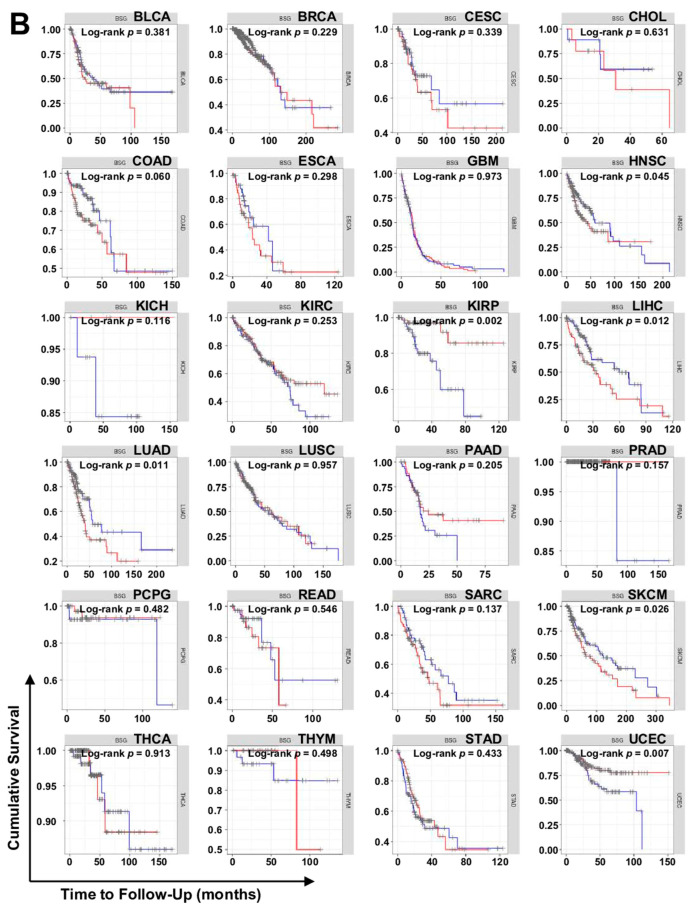
Correlation of *PPIA* and *BSG* expression on patient survival. (**A**) Relationship of *PPIA* mRNA expression. (**B**) Relationship of *BSG* mRNA expression. The data were obtained through TIMER analysis using the TCGA database. The full name of each carcinoma is described in the legend in Figure 5. Red line, high (top 25% expression group); blue line, low (bottom 25% expression group).

We further performed a survival analysis based on the expression of *PPIA* and *BSG* using Tumor Immune Estimation Resource (TIMER). The higher the expression of *PPIA*, the worse the survival in bladder carcinoma, esophageal carcinoma, kidney chromophobe, liver hepatocellular carcinoma, lung adenocarcinoma, pancreatic adenocarcinoma, sarcoma, skin cutaneous melanoma, and stomach adenocarcinoma (Figure 7A). The higher the expression of *BSG* in bladder carcinoma, cervical squamous cell carcinoma, liver hepatocellular carcinoma, lung adenocarcinoma, rectal adenocarcinoma, sarcoma, and skin cutaneous melanoma, the worse the patient survival (Figure 7B).

Interestingly, the expression levels of *PPIA* and *BSG* in lung squamous cell carcinoma did not affect patient survival, whereas the overexpression of *PPIA* and *BSG* decreased survival of patients with lung adenocarcinoma. These results indicate that the upregulation of *PPIA* and *BSG* expression may be implicated in the poorer prognosis of patients with lung adenocarcinoma compared with patients with lung squamous cell carcinoma. In addition, the survival of patients with liver hepatocellular carcinoma decreased with the overexpression of *PPIA* and *BSG*, suggesting that the inhibition of the CypA/CD147 axis may improve patient survival. Indeed, the anti-CD147 drug metuximab (Licartin) prevented tumor recurrence after orthotopic liver transplantation or percutaneous radiofrequency ablation in patients with advanced hepatocellular carcinoma and thus increased the survival rate [103,104]. These results support the clinical relevance of CypA/CD147 overexpression in the poor prognosis of patients with liver or lung cancer.

**Figure 8:**
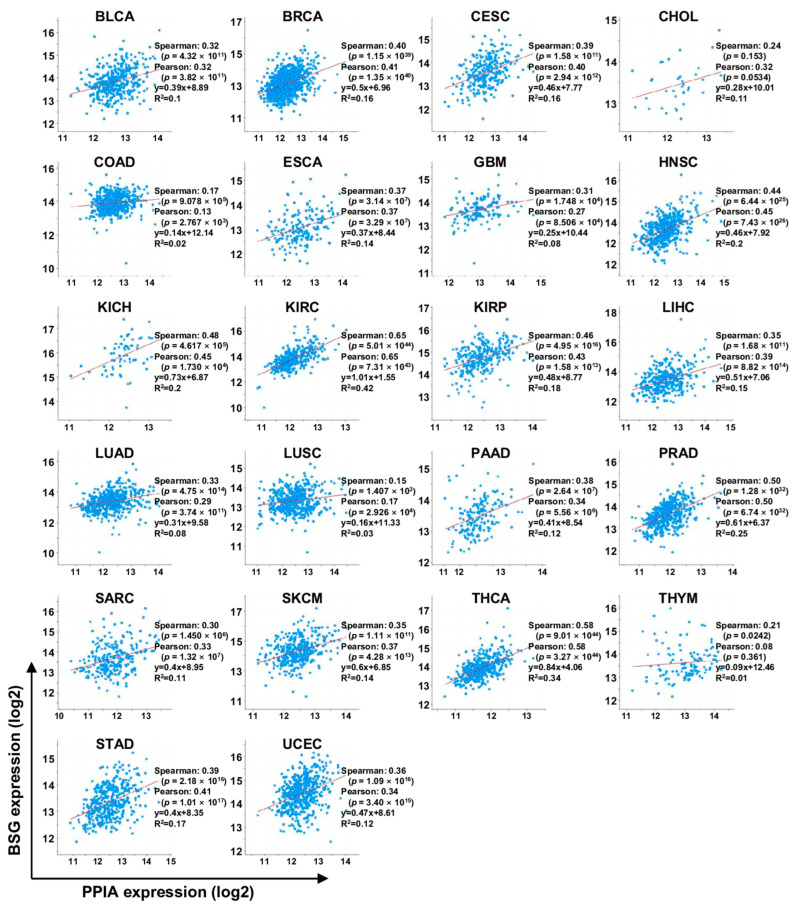
Correlation between transcription levels of *PPIA* and *BSG*. The data were obtained through cBioPortal analysis using the TCGA database. The full name of each carcinoma is described in the legend in Figure 5.

We also analyzed the correlation between *PPIA* and *BSG* expression in pan-cancer using cBioPortal. However, pheochromocytoma, paraganglioma, and rectal adenocarcinoma could not be diagnosed. The expression of *PPIA* and *BSG* was positively correlated in all 22 tumor types analyzed, indicating that the CypA/CD147 interaction plays a crucial role in cancer development (Figure 8). Collectively, our TCGA data analysis validated a significant association between *PPIA/BSG* overexpression and poor prognoses, such as low survival rate and high cancer stage, in several tumor types, including liver and lung cancers. This review suggests that CypA/CD147 may be a promising diagnostic and prognostic biomarker for certain cancers.

## 6. Conclusions and Future Perspectives

Despite remarkable advances in diagnostic and therapeutic strategies for cancer, the survival rate of patients with advanced or metastatic cancer remains low due to treatment resistance and recurrence. Many studies have demonstrated that CSCs are central drivers of cancer progression, metastasis, drug resistance, and relapse. Therefore, targeted therapies that can eradicate CSCs may improve clinical outcomes for patients with advanced cancer. This review highlights the critical roles of CypA/CD147 interactions in cancer pathology, therapeutics targeting CypA/CD147 signaling, and the value of the CypA/CD147 axis as a clinical biomarker. CypA/CD147 expression increases the survival and maintenance of CSCs in several cancer types by activating STAT3, Wnt/β-catenin, Notch, or PI3K/AKT signaling. Furthermore, inhibition of the CypA/CD147 axis sensitizes tumor cells to chemoradiotherapy by eliminating CSCs. Thus, targeting the CypA/CD147 interaction could be a promising strategy for effectively eradicating CSCs. However, further in vitro and in vivo studies are required to comprehensively understand the role of the CypA/CD147 axis in regulating the properties of CSCs to accumulate additional in-depth knowledge.

Clinical studies in the literature combined with our analysis using TCGA data have revealed that the mRNA expression levels of CypA and CD147 are higher in tumor tissues than in normal tissues of different tumor types. In addition, their overexpression is associated with poor prognosis in patients with several types of cancers. Clinically, CypA and CD147 expression is positively correlated in most cancer types. These results suggest that CypA and CD147 have the potential to be diagnostic and prognostic biomarkers for specific cases of cancer. However, the clinical relevance of CypA/CD147 interactions in aspects such as tumor subtype, tumor grade, and population-based cohort remains unclear. Therefore, acquiring additional clinical data and integrated interpretations based on multi-omics is necessary to resolve these questions.

## 7. TCGA Database Analysis

### 7.1. UALCAN Analysis

UALCAN (http://ualcan.path.uab.edu/index.html) is a web resource for analyzing the TCGA database. UALCAN can also be used to perform pan-cancer gene expression analysis and provides graphs and plots representing gene expression [109]. UALCAN was used to analyze the expression levels of *PPIA* and *BSG* in different tumor types and cancer stages (accessed on 13 July 2022). The threshold was *p* = 0.05.

### 7.2. TIMER Analysis

TIMER (https://cistrome.shinyapps.io/timer) provides analysis plots of immune cell invasion or gene transcription and survival time in the microenvironment of various types of cancer using the TCGA database [110]. TIMER was used to analyze the correlation between *PPIA* and *BSG* expression levels and cancer survival according to individual datasets (accessed on 12 July 2022).

### 7.3. cBioPortal Analysis

cBioPortal (https://www.cbioportal.org) enables large-scale data processing, statistical analysis, and graphical review of tumors from the gene to protein level using the TCGA database [111]. cBioPortal was used to analyze the correlation between *PPIA* and *BSG* expression according to cancer type (accessed on 12 July 2022).

## Figures and Tables

**Table 3:** The mRNA expression levels of *PPIA* in normal and tumor samples.

TCGA Samples	Expression of *PPIA*			
	Normal Samples		Tumor Samples	
	Number of Samples	Median Expression (Log2)	Number of Samples	Median Expression (Log2)
BLCA	19	6.985▼	408	7.691▲
BRCA	114	7.077▼	1097	7.572▲
CESC	3	6.748▼	305	7.728▲
CHOL	9	5.485▼	36	7.41▲
COAD	41	7.013▼	286	7.756▲
ESCA	11	6.031▼	184	7.842▲
GBM	5	7.263▼	156	8.328▲
HNSC	44	6.78▼	520	7.474▲
KICH	25	7.143▼	67	7.217▲
KIRC	72	7.074▼	533	7.27▲
KIRP	32	6.807▼	290	7.504▲
LIHC	50	5.672▼	371	6.883▲
LUAD	59	6.89▼	515	7.387▲
LUSC	52	6.916▼	503	7.613▲
PAAD	4	7.009▼	178	7.178▲
PRAD	52	6.729▼	497	7.035▲
PCPG	3	6.686▼	179	6.994▲
READ	10	7.106▼	166	8.21▲
SARC	2	6.672▼	260	7.044▲
SKCM	1	7.477▼	472	7.662▲
THCA	59	6.85▼	505	6.876▲
THYM	2	7.393▼	120	8.092▲
STAD	34	6.592▼	415	7.623▲
UCEC	35	6.905▼	546	8.881▲

**Table 4:** The mRNA expression levels of *BSG* in normal and tumor samples.

TCGA Samples	Expression of *BSG*			
	Normal Samples		Tumor Samples	
	Number of Samples	Median Expression (Log2)	Number of Samples	Median Expression (Log2)
BLCA	19	9.097▼	408	9.437▲
BRCA	114	8.505▼	1097	9.027▲
CESC	3	9.126▼	305	9.276▲
CHOL	9	6.909▼	36	9.00▲
COAD	41	10.581▲	286	9.653▼
ESCA	11	8.682▼	184	9.39▲
GBM	5	9.608▲	156	9.575▼
HNSC	44	8.455▼	520	9.286▲
KICH	25	10.039▼	67	11.292▲
KIRC	72	9.674▲	533	9.535▼
KIRP	32	10.062▼	290	10.475▲
LIHC	50	6.894▼	371	8.409▲
LUAD	59	8.916▼	515	9.144▲
LUSC	52	8.76▼	503	9.033▲
PAAD	4	9.01▼	178	9.12▲
PRAD	52	9.073▼	497	9.368▲
PCPG	3	9.959▼	179	10.077▲
READ	10	9.925▲	166	9.487▼
SARC	2	9.879▲	260	9.384▼
SKCM	1	9.364▼	472	10.145▲
THCA	59	9.421▼	505	9.61▲
THYM	2	8.774▼	120	9.056▲
STAD	34	9.015▼	415	9.514▲
UCEC	35	9.347▼	546	10.024▲

## Data Availability

All data are contained within the manuscript. The TCGA database is available in a publicly accessible repository.
